# Psychiatryteleconsultation/teleconsulting: Experience of general confinement in Tunisia

**DOI:** 10.1192/j.eurpsy.2021.1745

**Published:** 2021-08-13

**Authors:** R. Jomli, H. Jemli, U. Ouali, Y. Zgueb, F. Nacef

**Affiliations:** 1 Faculty Of Medicine Of Tunis, university of tunis elmanar, tunis, Tunisia; 2 Avicenne, manouba psychiatry, manouba, Tunisia

**Keywords:** Tunisia, Psychiatryteleconsultation, COVID-19, general confinement

## Abstract

**Introduction:**

The outbreak of the Covid-19 epidemic in Tunisia has led a total confinement startingfrom March 23rd, 2020. Remote interventions of psychiatrists and psychologists have been set up to help tunisiancitizens. The requestsconcerned panic attacks, acute stress disorder, sleepdisorders and relapse of some patients followed for mooddisorders, obsessive compulsive disorder or anxietydisorders.

**Objectives:**

We propose to present 3 types of interventions by apsychiatrist in response to the request of 3 Tunisiancitizensin this first experience.

**Methods:**

We will summarize three interventions with the history of each patient and his or herrequest as well as the short term evolution of the presenteddisorder.

**Results:**

As a first experience, weresponded to numerousrequestsfrom people of all ages and living all over Tunisia. The first intervention concerned a lady whowasvolontarilyvomiting in order to maintainhercurrentweight.Shewasafraid of having the covid infectionrelated to a sore throat. The second situation is a gentleman whodid not toleratehome confinement because for 20 yearshe has been going out for adailywalk at exactly 4 pm. The thirdrequest came from a father living in a rural area who chose to self isolate in his room and refused to go out and seehischildrendespitetheir pressing demand. We offered active listening, reassurance and behavioural psychotherapy techniques.

**Conclusions:**

Duringthis first experience in Tunisia, wefoundthatcitizens haveadhered to new communication techniques. Anxiety, stress and relapse of priormedical conditions were the mostfrequent diagnoses. Several interventions have proven to be effective despite obstacles related to teleconsultation.

**Disclosure:**

No significant relationships.

